# Supplementary material for: Immunogenicity and efficacy of VLA2001 vaccine against SARS-CoV-2 infection in male cynomolgus macaques
Source: Commun Med (Lond). 2024 Apr 3;4:62. doi: 10.1038/s43856-024-00488-w (PMC10991505; doi:10.1038/s43856-024-00488-w)
Supplement: Supplementary file 2 — Supplementary Information [file 43856_2024_488_MOESM2_ESM.pdf]

Supplementary Table 1. Characteristics of the animals included into the study.

ID	Experimental group	Sexe	Age at 1st immunization (months)	Weight at 1st immunization (kg)	Necropsy (days post exposure)	Symbol and color for figures
MF1	Ctrl	male	46.3	5.11	15	●
MF2	Ctrl	male	45.0	3.82	7	■
MF3	Ctrl	male	44.6	4.74	15	▲
MF4	Ctrl	male	44.7	3.67	7	▼
MF5	Ctrl	male	44.3	4.48	15	◆
MF6	Ctrl	male	45.5	4.32	15	⬢
MF7	Ctrl	male	44.5	5.50	7	×
MF8	Ctrl	male	43.8	3.89	7	+
MF9	High	male	46.2	4.46	15	●
MF10	High	male	45.8	4.58	7	■
MF11	High	male	46.1	5.00	7	▲
MF12	High	male	45.0	6.35	15	▼
MF13	High	male	45.1	4.25	7	◆
MF14	High	male	44.3	4.00	15	⬢
MF15	High	male	44.7	3.07	15	×
MF16	High	male	44.2	4.00	7	+
MF17	Medium	male	46.2	6.00	15	●
MF18	Medium	male	46.2	5.60	15	■
MF19	Medium	male	45.7	5.85	7	▲
MF20	Medium	male	44.8	3.28	15	▼
MF21	Medium	male	44.7	4.79	15	◆
MF22	Medium	male	45.1	4.82	7	⬢
MF23	Medium	male	44.5	4.23	7	×
MF24	Medium	male	43.7	4.60	7	+

Supplementary Table 2. Histopathological score. Histopathology scoring system used for SARS-CoV-2 induced pulmonary lesions.

Parameters	Score	Description
Septal cellularity	0	Normal alveolar wall (AW)
	1	1-2 red blood cell (RBC), thick AW
	2	2-4 RBC, thick AW
	3	5-10 RBC, thick AW
	4	>10, thick AW
Septal fibrosis	0	Absent
	1	Present
Type II pneumocytes hyperplasia	0	Absent
	1	Present
Alveolar macrophages	0	Absent
	1	1-5 per x20 field of view (FOV)
	2	6-10 per x20 FOV
	3	11-15 per x20 FOV
	4	>15 per x20 FOV
Alveolar neutrophils	0	Absent
	1	1-5 per x20 FOV
	2	6-10 per x20 FOV
	3	11-15 per x20 FOV
	4	>15 per x20 FOV

Supplementary Figure 1. Antibody responses in cynomolgus macaques. Individual titers (AU/mL) of IgG binding to SARS-CoV-2 S (upper), RBD (center) and N (lower) proteins measured in sera samples. BL: baseline approximatively 3 weeks before first immunization. Black dotted vertical lines represent vaccinations (weeks 0 and 3). Red dotted vertical lines represent SARS-CoV-2 exposure (week 7).

Each animal is identified by distinct symbols (Supplementary Table 1 for matching symbols). Control group is in black, High dose group is in blue and Medium dose group is in orange.

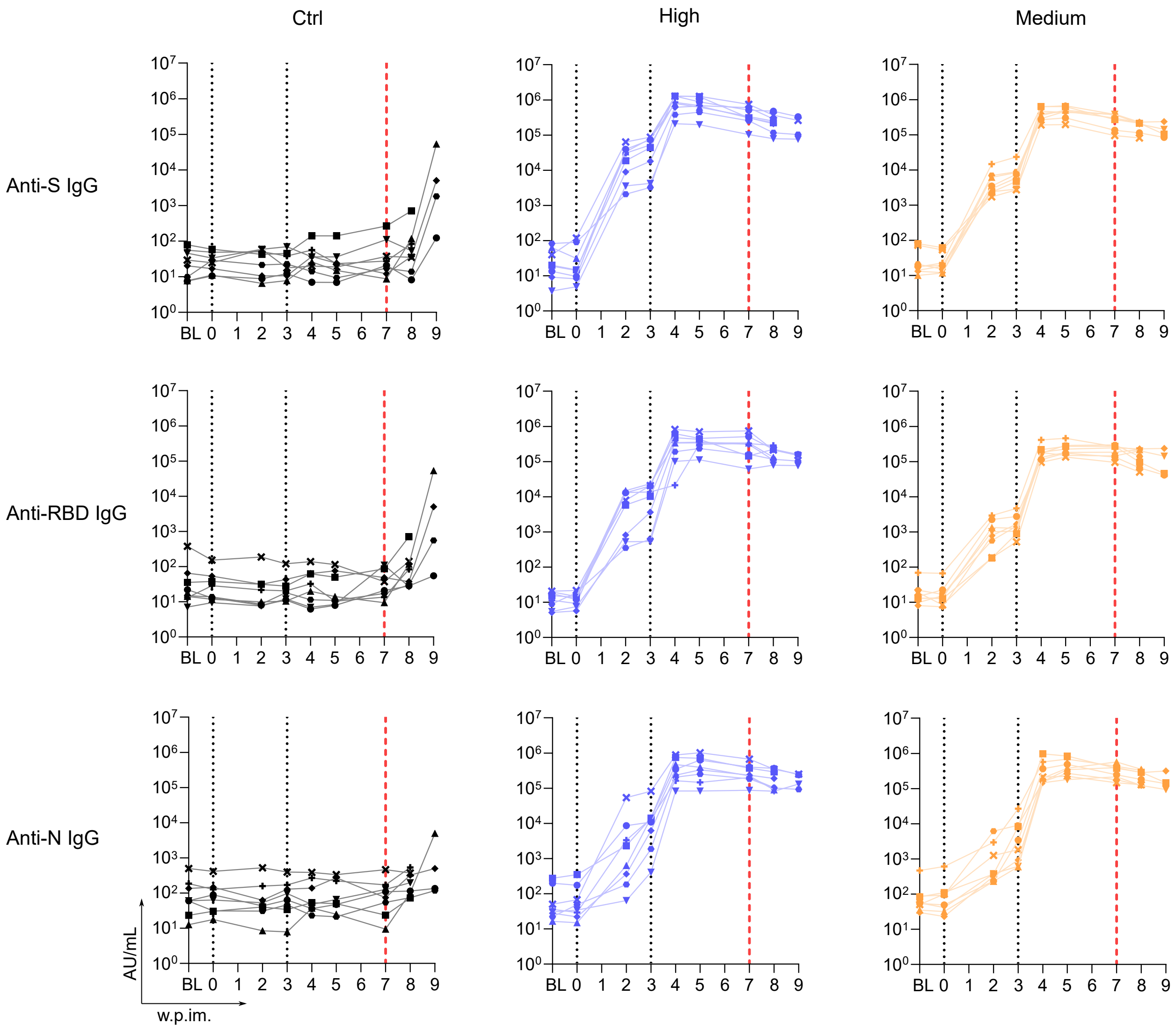

Ctrl
High
Medium
Anti-S IgG
Anti-RBD IgG
Anti-N IgG
10^0
10^1
10^2
10^3
10^4
10^5
10^6
10^7
BL 0 1 2 3 4 5 6 7 8 9
AU/mL
w.p.im.

Supplementary Figure 2. Neutralizing antibody responses in cynomolgus macaques. Individual titers of neutralizing antibody to SARS-CoV-2 (NT_{50}: 50 % neutralizing titer) in sera samples. BL: baseline approximatively 3 weeks before first immunization. Black vertical dotted lines represent vaccinations (weeks 0 and 3). Red vertical dotted lines represent SARS-CoV-2 exposure (week 7). Graph starts at the limit of quantification at 5. Dotted horizontal line indicates the titer of the WHO International Standard for anti-SARS-CoV-2 immunoglobulin in human determined in the same assay.

Each animal is identified by distinct symbols (Supplementary Table 1 for matching symbols). Control group is in black, High dose group is in blue and Medium dose group is in orange.

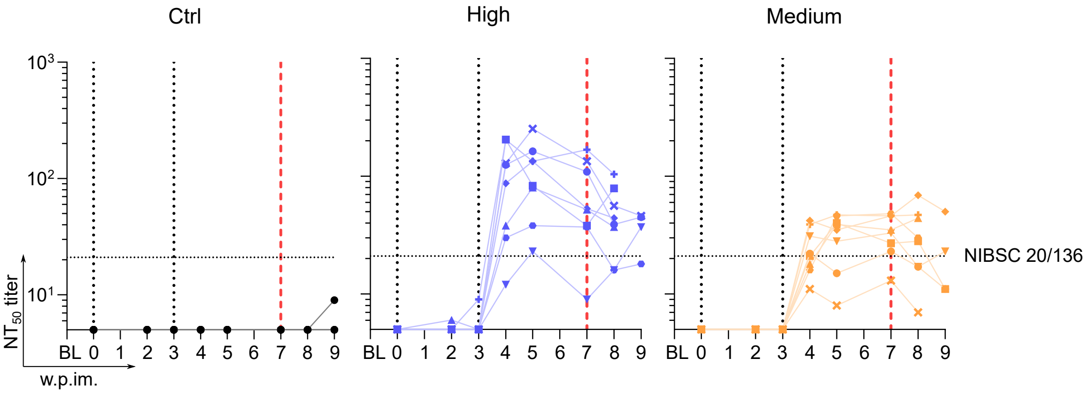
Ctrl
High
Medium
10^3
10^2
10^1
NT_{50} titer
w.p.im.
BL 0 1 2 3 4 5 6 7 8 9
NIBSC 20/136

Supplementary Figure 3. Subgenomic viral quantification (sgRNA) in fluids from respiratory tract following SARS-CoV-2 exposure. a, Peak of subgenomic viral RNA (sgRNA) quantification in nasopharyngeal and tracheal fluids collected with swabs (Box and whiskers, min to max) (non-parametric Kruskal-Wallis and Dunn's multiple comparisons tests; n=8 per group). Dotted horizontal line represents the lower limit of quantification (LLOQ) at 3.87 log_{10} copies/mL. Graphs start at the lower limit of detection (LLOD) at 2.87 log_{10} copies/mL. Values below the LLOD were plotted at 2.87 log_{10} copies/mL. **b,** Individual sgRNA quantification in fluids from bronchoalveolar lavages (BAL) performed at day 3 post SARS-CoV-2 exposure (Box and whiskers, min to max) (non-parametric Kruskal-Wallis and Dunn's multiple comparisons tests; n=8 per group). Dotted horizontal line represents the LLOQ at 3.87 log_{10} copies/mL. Graph starts at the LLOD at 2.87 log_{10} copies/mL. Values below the LLOD were plotted at 2.87 log_{10} copies/mL.

Each animal is identified by distinct symbols (Supplementary Table 1 for matching symbols). Control group is in black, High dose group is in blue and Medium dose group is in orange.

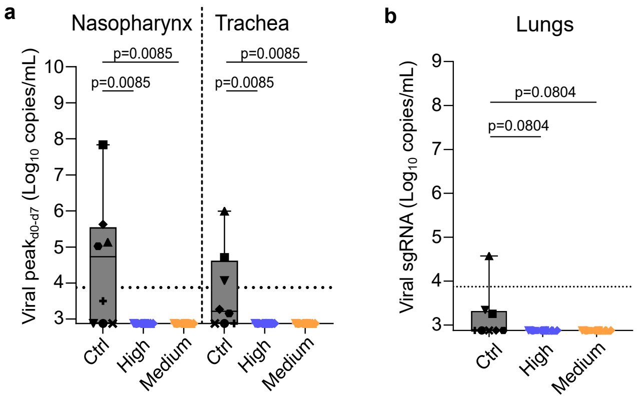
a
Nasopharynx
Trachea
Viral peak d0-d7 (Log10 copies/mL)
p=0.0085
p=0.0085
p=0.0085
p=0.0085
Ctrl
High
Medium
Ctrl
High
Medium
b
Lungs
Viral sgRNA (Log10 copies/mL)
p=0.0804
p=0.0804
Ctrl
High
Medium

Supplementary Figure 4. Illustrative images of CT acquisition following SARS-CoV-2 exposure. Representative transversal slices of chest CT scans performed at baseline (BL, week 5 post first immunization), day 3 and day 10 post SARS-CoV-2 exposure showing typical lung lesions.

Control group is in black, High dose group is in blue and Medium dose group is in orange.

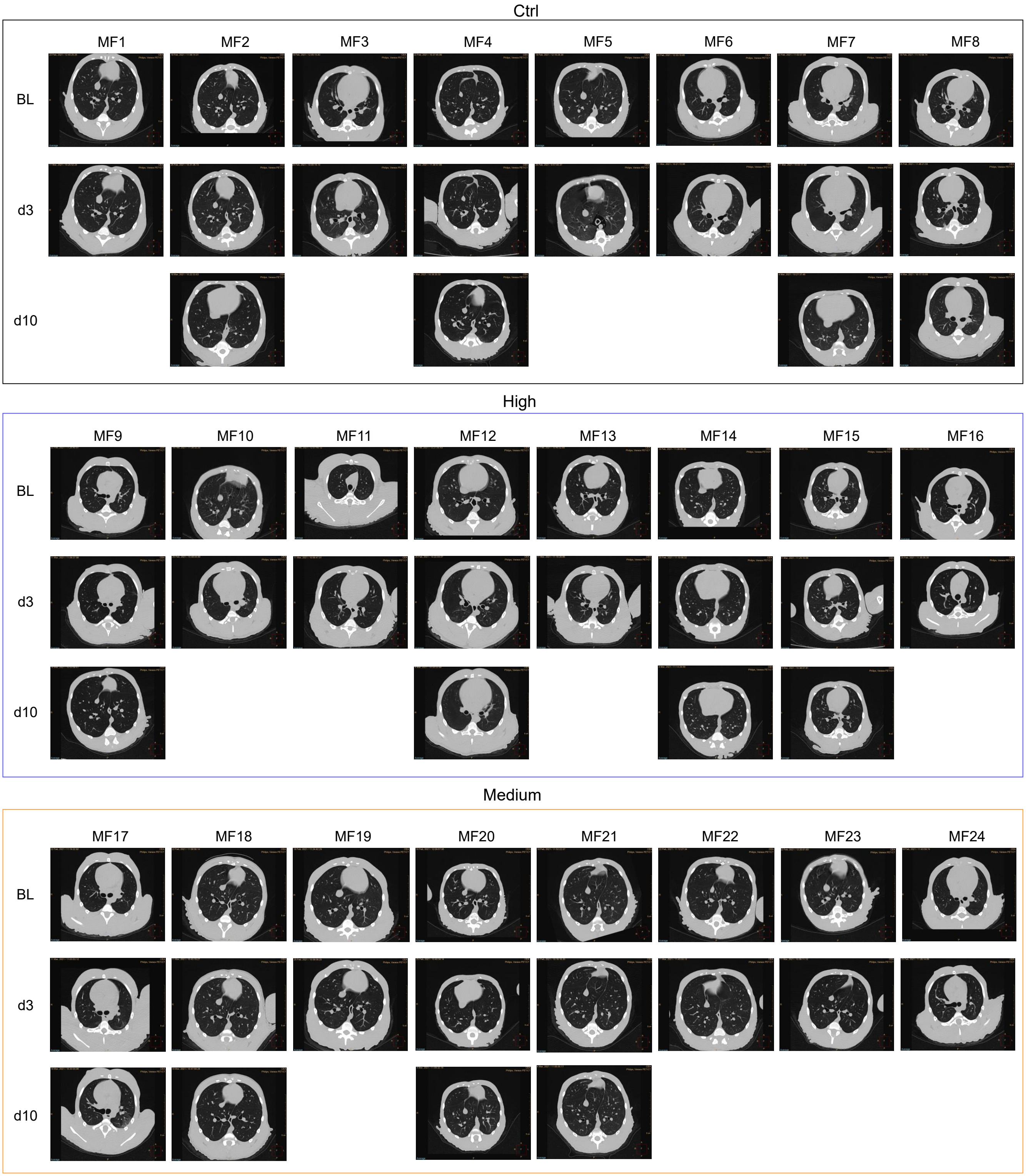
Ctrl
MF1
MF2
MF3
MF4
MF5
MF6
MF7
MF8
BL
d3
d10
High
MF9
MF10
MF11
MF12
MF13
MF14
MF15
MF16
BL
d3
d10
Medium
MF17
MF18
MF19
MF20
MF21
MF22
MF23
MF24
BL
d3
d10
