# Supplementary material for: Immunogenicity and efficacy of VLA2001 vaccine against SARS-CoV-2 infection in male cynomolgus macaques
Source: Commun Med (Lond). 2024 Apr 3;4:62. doi: 10.1038/s43856-024-00488-w (PMC10991505; doi:10.1038/s43856-024-00488-w)
Supplement: Supplementary file 3 — Description of Additional Supplementary Files [file 43856_2024_488_MOESM3_ESM.pdf]

Description of Additional Supplementary Files

File Name: Supplementary Data 1

Description: Raw data are included with a summary and one sheet per type of raw data. Each sheet is labelled by the figure number and panel.
