# Supplementary material for: Immunogenicity and efficacy of VLA2001 vaccine against SARS-CoV-2 infection in male cynomolgus macaques
Source: Commun Med (Lond). 2024 Apr 3;4:62. doi: 10.1038/s43856-024-00488-w (PMC10991505; doi:10.1038/s43856-024-00488-w)
Supplement: Supplementary file 5 — Reporting summary [file 43856_2024_488_MOESM5_ESM.pdf]

nature portfolio

Corresponding author(s): Roger Le Grand

Last updated by author(s): Feb 16, 2024

Reporting Summary

Nature Portfolio wishes to improve the reproducibility of the work that we publish. This form provides structure for consistency and transparency in reporting. For further information on Nature Portfolio policies, see our Editorial Policies and the Editorial Policy Checklist.

Statistics

For all statistical analyses, confirm that the following items are present in the figure legend, table legend, main text, or Methods section.

n/a	Confirmed	
☐	☒	The exact sample size (*n*) for each experimental group/condition, given as a discrete number and unit of measurement
☐	☒	A statement on whether measurements were taken from distinct samples or whether the same sample was measured repeatedly
☐	☒	The statistical test(s) used AND whether they are one- or two-sided<br>*Only common tests should be described solely by name; describe more complex techniques in the Methods section.*
☒	☐	A description of all covariates tested
☐	☒	A description of any assumptions or corrections, such as tests of normality and adjustment for multiple comparisons
☐	☒	A full description of the statistical parameters including central tendency (e.g. means) or other basic estimates (e.g. regression coefficient) AND variation (e.g. standard deviation) or associated estimates of uncertainty (e.g. confidence intervals)
☐	☒	For null hypothesis testing, the test statistic (e.g. F, t, r) with confidence intervals, effect sizes, degrees of freedom and P value noted<br>*Give P values as exact values whenever suitable.*
☒	☐	For Bayesian analysis, information on the choice of priors and Markov chain Monte Carlo settings
☒	☐	For hierarchical and complex designs, identification of the appropriate level for tests and full reporting of outcomes
☒	☐	Estimates of effect sizes (e.g. Cohen's *d*, Pearson's *r*), indicating how they were calculated

Our web collection on statistics for biologists contains articles on many of the points above.

Software and code

Policy information about availability of computer code

Data collection	Data were collected using classical Excel files. Animals data were stored in a in-house Laboratory Information Management System, called Batlab.
Data analysis	Data were analyzed using GraphPad Prism v8, GraphPad Prism v9, FlowJo v10, AID ELISpot v8, CFX Maestro and IntelliSpace Portal v8.

For manuscripts utilizing custom algorithms or software that are central to the research but not yet described in published literature, software must be made available to editors and reviewers. We strongly encourage code deposition in a community repository (e.g. GitHub). See the Nature Portfolio guidelines for submitting code & software for further information.

Data

Policy information about availability of data

All manuscripts must include a data availability statement. This statement should provide the following information, where applicable:

- Accession codes, unique identifiers, or web links for publicly available datasets
- A description of any restrictions on data availability
- For clinical datasets or third party data, please ensure that the statement adheres to our policy

Data that support the findings of this study are provided in the Supplementataty Data file uploaded with the manuscript.

Research involving human participants, their data, or biological material

Policy information about studies with human participants or human data. See also policy information about sex, gender (identity/presentation), and sexual orientation and race, ethnicity and racism.

Reporting on sex and gender	No human participants were included in this study.
Reporting on race, ethnicity, or other socially relevant groupings	No human participants were included in this study.
Population characteristics	No human participants were included in this study.
Recruitment	No human participants were included in this study.
Ethics oversight	No human participants were included in this study.

Note that full information on the approval of the study protocol must also be provided in the manuscript.

Field-specific reporting

Please select the one below that is the best fit for your research. If you are not sure, read the appropriate sections before making your selection.

☒ Life sciences ☐ Behavioural & social sciences ☐ Ecological, evolutionary & environmental sciences

For a reference copy of the document with all sections, see nature.com/documents/nr-reporting-summary-flat.pdf

Life sciences study design

All studies must disclose on these points even when the disclosure is negative.

Sample size	Sample size was determined as the minimal number allowing non-parametric statistical analysis while complying with the 3Rs rule on reducing, replacing and refining the use of animals for scientific purpose.
Data exclusions	No data has been excluded from analysis.
Replication	Replicates were performed for all measurements within each assay (Duplicates for RT-qPCR, ELISA and ELISpot).
Randomization	The cynomolgus macaques were randomly assigned, with RAND function of Excel software, into three experimental groups.
Blinding	For security reason, animal ID and experimental group are indicated on the housing cage, thus Animals care, clinical examination and sampling was not blinded because constrains associated to BSL3 containement.<br>Cynomolgus macaque viral loads, CT scoring, IFN-γ ELISpot, ICS, quantification of SARS-CoV-2 antibodies and quantification of antibody-induced inhibition of ACE-2 binding were determined blinded.

Reporting for specific materials, systems and methods

We require information from authors about some types of materials, experimental systems and methods used in many studies. Here, indicate whether each material, system or method listed is relevant to your study. If you are not sure if a list item applies to your research, read the appropriate section before selecting a response.

Materials & experimental systems

n/a	Involved in the study	
☐	☒	Antibodies
☐	☒	Eukaryotic cell lines
☒	☐	Palaeontology and archaeology
☐	☒	Animals and other organisms
☒	☐	Clinical data
☒	☐	Dual use research of concern
☒	☐	Plants

Methods

n/a	Involved in the study	
☒	☐	ChIP-seq
☐	☒	Flow cytometry
☒	☐	MRI-based neuroimaging

Antibodies

Antibodies used	Anti-IL-2-PerCP-Cy5.5 (MQ1-17H12, 560708, 0058021, Becton Dickinson - dil. 1:10)

Antibodies used	Anti-IL-17a-AF700 (N49-653, 560613, 0156497, Becton Dickinson - dil. 1:20)<br>Anti-IFN-γ-V450 (B27, 560371, 9274224, Becton Dickinson - dil. 1:33.3)<br>Anti-IFN-γ-V450 (B27, 560371, 7069869, Becton Dickinson - dil. 1:33.3)<br>Anti-TNF-α-BV605 (Mab11, 502936, B298444, BioLegend - dil. 1:30.3)<br>Anti-IL-13-BV711 (JES10-5A2, 564288, 0309149, Becton Dickinson - dil. 1:20)<br>Anti-CD69-PE (FN50, 310906, B324050, BioLegend - dil. 1:10)<br>Anti-CD69-PE (FN50, 310906, B313144, BioLegend - dil. 1:10)<br>Anti-CD137-APC (4B4-1, 550890, 8061835, Becton Dickinson - dil. 1:20)<br>Anti-CD137-APC (4B4-1, 550890, 9287986, Becton Dickinson - dil. 1:20)<br>Anti-CD154-FITC (TRAP1, 555699, 8032873, Becton Dickinson - dil. 1:20)<br>Anti-CD3-APC-Cy7 (SP34-2, 557757, 9252411, Becton Dickinson - dil. 1:200)<br>Anti-CD4-BV510 (L200, 563094, 195414, Becton Dickinson - dil. 1:33,3)<br>Anti-CD8-PE-Vio770 (BW135/80, 130-113-159, 5210208846, Miltenyi Biotec - dil. 1:50)<br>Anti-CD8-PE-Vio770 (BW135/80, 130-113-159, 5201207189, Miltenyi Biotec - dil. 1:50)<br>Anti-CD28/49d (347690, 9315490, Becton Dickinson)<br>Anti-CD28/49d (347690, 253692, Becton Dickinson)<br>Anti-CD28/49d (347690, 316920, Becton Dickinson)<br>Recombinant anti-SARS-CoV-2 Spike Glycoprotein S1 (CR3022, ab273073, GR3348907-1, Abcam)<br>Anti-human IFN-γ mAb ALP (n/a, 7-B6-1, 43746, Mabtech)<br>Anti-human IFN-γ mAb ALP (n/a, 7-B6-1, 43454, Mabtech)<br>Spike RBD mAb clone 43 - mouse IgG1 (Sino Biological, 40591-MM43, Lot: MA14JU1803)<br>CR3022 SARS-CoV-2 mAb - mouse IgG2b (Absolute Antibody, Ab01680-3.0, Lot: T2014B02)<br>Goat anti-mouse IgG-HRP (Jackson Immuno, 115-036-062 , Lot: 89024)<br>Goat anti-mouse IgG1-HRP (Jackson Immuno, 115-035-205, Lot: 148255)<br>Goat anti-mouse IgG2a-HRP (Jackson Immuno, 115-035-206, Lot: 147741)
Validation	See the corresponding manufacturer datasheets on webpages for validation. Crossreactivity of antibodies used in NHP experiments were confirmed using the "NHP reagents" online database : https://www.nhpreagents.org/ReactivityDatabase or on manufacturer datasheets in "reactivity " section.

Eukaryotic cell lines

Policy information about cell lines and Sex and Gender in Research

Cell line source(s)	JCRB Cell Bank (National Institutes of Biomedical Innovation, Health and Nutrition) ; e.Enzyme
Authentication	VeroE6/TMPRSS2 ; HEK293-hACE2
Mycoplasma contamination	No ; No
Commonly misidentified lines (See ICLAC register)	No commonly misidentified cell lines were used in this study.

Animals and other research organisms

Policy information about studies involving animals; ARRIVE guidelines recommended for reporting animal research, and Sex and Gender in Research

Laboratory animals	Six to eight-weeks old female BALB/c mice and 43.8 to 46.3-months old male cynomolgus macaques were included.
Wild animals	No wild animals were used in this study.
Reporting on sex	No sex-based analysis were performed.
Field-collected samples	No field-collected samples were used in this study.
Ethics oversight	For mice, experiments were conducted in accordance with Austrian law (BGBl No. 114/2012) and approved by the appropriate local authorities. Experimental procedures were reviewed and approved by Valneva's animal welfare committee.<br>For non-human primates, experiments were performed in compliance with European Directive 2010/63/EU, the French regulations and the Standards for Human Care and Use of Laboratory Animals of the Office for Laboratory Animal Welfare (OLAW, assurance number #A5826-01, US). The protocols were approved by the institutional ethical committee “Comité d’Ethique en Expérimentation Animale du Commissariat à l’Energie Atomique et aux Energies Alternatives” (CEtEA #44) under statement number A20-037. The study was authorized by the Research, Innovation and Education Ministry under registration number APAFIS#24434-2020030216532863v3.

Note that full information on the approval of the study protocol must also be provided in the manuscript.

Flow Cytometry

Plots

Confirm that:

☒ The axis labels state the marker and fluorochrome used (e.g. CD4-FITC).

☒ The axis scales are clearly visible. Include numbers along axes only for bottom left plot of group (a 'group' is an analysis of identical markers).

☒ All plots are contour plots with outliers or pseudocolor plots.

☒ A numerical value for number of cells or percentage (with statistics) is provided.

Methodology

Sample preparation	One million PBMCs were cultured in complete medium (RPMI1640 Glutamax+, Gibco; supplemented with 10 % FBS), supplemented with co-stimulatory antibodies (FastImmune CD28/CD49d, Becton Dickinson), and stimulated with S, RBD or N sequence overlapping peptide pools as above at a final concentration of 2 µg/mL. Brefeldin A was added to each well at a final concentration of 10 µg/mL and the plate was incubated at 37 °C, 5 % CO2 for 18 hours. Next, cells were washed, stained with a viability dye (LIVE/DEAD fixable Blue dead cell stain kit, ThermoFisher), and then fixed and permeabilized (Cytofix/Cytoperm, Becton Dickinson). Fixed and permeabilized cells were stored at -80 °C before the staining procedure. After thawing of fixed and permeabilized cells, antibody staining was performed. After 30 minutes of incubation at 4 °C in the dark, cells were washed in BD Perm/Wash buffer and then acquired on the ZE5 flow cytometer (Becton Dickinson).
Instrument	LSRII cytometer (Becton Dickinson).
Software	FlowJo v10 software.
Cell population abundance	No cell-sorting was performed in this study.
Gating strategy	First gating on "time" VS SSC-H was performed, then singlet were selected on SSC-W vs SSC-A gating. Alive cells were selected on SSC-A vs viability marker. Then lineage markers were used to analyze cell subset of interest. The gating strategy as already published in Marlin et al. (reference 34 in the manuscript)

☐ Tick this box to confirm that a figure exemplifying the gating strategy is provided in the Supplementary Information.
